# Psychological Influence of Self-Management on Exercise Self-Confidence, Satisfaction, and Commitment of Martial Arts Practitioners in Korea: A Meta-Analytic Approach

**DOI:** 10.3389/fpsyg.2021.691974

**Published:** 2021-05-31

**Authors:** Hyun-Duck Kim, Angelita Bautista Cruz

**Affiliations:** ^1^Department of Sport Marketing, Keimyung University, Daegu, South Korea; ^2^Department of Physical Education, Keimyung University, Daegu, South Korea

**Keywords:** martial arts, self-regulation, psychological state, psychological skills training, Korean athletes

## Abstract

This study aimed to meta-analyze the relationship between self-management and exercise self-confidence, satisfaction, and commitment in both modern and traditional martial arts among Korean practitioners. We examined the level of sports participation and different martial arts sports as potential moderating variables. In total, 22 studies yielded 299 individual effect sizes and were included in the final meta-analytic pool. The analyses revealed a moderate effect of self-management on exercise satisfaction and self-confidence; and a large effect self-management on exercise commitment. Especially, the effect of the training dimension of self-management was large on exercise commitment and satisfaction, while that of the mental dimension was large on exercise self-confidence. The effects of self-management on exercise satisfaction, commitment, and self-confidence were large in judo, Ssireum, and wrestling, respectively. Finally, the use of self-management was relatively more effective for non-elite participants than for elite practitioners. Our results highlight the effectiveness of self-management to enhance Korean martial arts practitioners’ exercise self-confidence, commitment, and satisfaction, findings that may potentially be extended internationally and to other types of sports; further, they showcase the importance of the promotion of interventions and educational programs on how to incorporate/employ self-management in athletes’ sports training.

## Introduction

Athletes are often exposed to various stressors either during training or competition (Sarkar and Fletcher, 2014). These stressors may come from many psychological, environmental, physical, and other internal/external sources. Athletes’ stressors may originate from performance achievement, health concerns, weather, relationship, or leadership issues with coaches and peers (Hanton et al., 2005; Evans et al., 2012; Sarkar and Fletcher, 2014). In competitive martial arts in particular, stressors such as injuries, poor fitness condition, weight, subpar performances, and coach/teammates-athlete relationship concerns, physical pain, and cognitive/somatic state anxieties are normally encountered (Massey et al., 2015; Cintineo and Arent, 2019; Son et al., 2020). If athletes perceive these stressful demands to be threatening and with no capability to control the strains placed on them, it would lead them to negative physical and psychological responses (Fletcher and Sarkar, 2012) resulting in poor performance outcomes and psychological breakdown (Papacosta et al., 2016; Cintineo and Arent, 2019). It is therefore important for athletes to be aware of these stressful situations and have the appropriate skills or strategies on how to properly regulate undesirable thoughts, feelings, and behavioral reactions to stress that may negatively influence performance (Green and Svinth, 2010; Arnold et al., 2017).

Self-management is a concept used in many fields of study (Lorig and Holman, 2003; Gerhardt, 2007; Grady and Gough, 2014; Jung and Takeuchi, 2018) including sports. In the sports field, this term—which is analogous to self-regulation—is defined as people’s capacity to effectively monitor, control, and manage thoughts, emotions, and behaviors that could facilitate goal accomplishment (Weinberg and Gould, 2015; Furlonger et al., 2017). The goals may be monitoring physical development, regulating anxiety and arousal levels, or developing focus and concentration. If these goals are accomplished, in turn, may assist athletes in enhancing their positive psychological state and/or sports performance.

Generally, athletes have various self-management strategies at their disposal during periods of training and/or competition; for example, goal setting, arousal regulation, coping, mental training, self-confidence, and motivation (Weinberg and Gould, 2015; Arnold et al., 2017; Furlonger et al., 2017), all of which can enhance athletes’ control over the mental, emotional, technical, and physical dimensions of their sports performance. Most training programs typically have a set of pre-established goals. This goal may be improvement of tactical offense/defense movements through simulation training (Massey et al., 2015) or incorporating breathing techniques or positive self-talk to manage arousal and anxiety levels (Hatzigeorgiadis et al., 2011). Thus, we can infer that programs which integrate self-management strategy learning/practice with sports training can allow for the enhancement of athletes’ self-monitoring, -evaluating, and -reinforcing psychological and physiological skills related to essential facets of their sports performance. These learned strategies, thereby, allow athletes to modify their behaviors toward gaining desired outcomes (Critchfield and Vargas, 1991; Polaha et al., 2004; Collins and Durand-Bush, 2014; Furlonger et al., 2017). That is, athletes with well-developed self-management skills are capable to initiate, modify, and complete sport-related tasks (i.e., which require the development of higher levels of sports performance) with confidence, thereby making them less dependent on coaches’/sports psychologists’ constant instruction/support to achieve higher levels of sports performance (Im et al., 2015).

Concurring with this inference, studies have shown that the application of self-management strategy learning programs (i.e., to teach athletes how to utilize such strategies) can effectively improve the performance and psychological states of athletes in different sporting situations. For example, Vesković et al. (2019) investigated the effects of two psychological strategies on anxiety and self-confidence levels in Karate athletes and found improved self-confidence and decreased cognitive anxiety among elite Karate players who underwent modified autogenic and imagery trainings compared to those who did not. O’Brien et al. (2009), on the other hand, examined whether a goal-setting program could improve sports performance behaviors, competition outcomes, competitive anxiety, and self-confidence of elite and non-elite boxers; they found that, during and after the implementation of the goal-setting program, the boxers showed the following outcomes: improvements in their success rates of landed and defended punches; in their percentage of fights won; in their self-confidence; and their experienced anxieties were interpreted as facilitative rather than debilitative. Schwab Reese et al. (2012) endeavored to review the effectiveness of different self-management strategies to increase the self-management of injured athletes; they reported that imagery, goal-setting, and relaxation were useful to decrease negative psychological consequences, re-injury anxiety, and increase coping.

Summarizing, these citations underpin the effectiveness of self-management to facilitate positive outcomes in athletes’ performance and mental state in the sports field. These outcomes find consonance in Bandura’s self-efficacy theory (Bandura, 1977, 1997), which assumes that people’s beliefs can influence their ability to perform a behavior; nonetheless, the theory also remarks that the belief of being able to successfully perform a behavior depends on the strength of that belief and on the level of conduciveness of the environment. Accordingly, athletes with high beliefs in their self-management ability (e.g., to control their negative thoughts; plan their trainings to improve their physical and tactical skills; regulate their anxiety levels; and manage positive relationships with others on their own; etc.) may feel more confident to pursue behaviors based on specific goals. In this regard, a study showed that successfully achieving goals while employing self-management strategies could enhance athletes’ self-confidence (i.e., their beliefs on themselves; Dishman et al., 2005). Deci and Ryan (1985)’s self-determination theory is another approach to explain athletes’ positive sport performance through self-management. This theory claims that self-determined types of motivational regulations will affect behavior if the three basic psychological factors of relatedness, competence, and autonomy are more or less satisfied. It also suggests that depending on the level of satisfaction from the three psychological needs, different cognitive, affective and behavioral outcomes will occur. Moreover, autonomy and competence are found to be associated with self-regulation (Deci and Ryan, 2000; Guillet et al., 2002). Following these points in martial arts, if athletes (e.g., Korean athletes) participate in sport for their own pleasure and satisfaction (autonomy), personal goals achievement (competence), and team support/acknowledgment (relatedness), their motivation to perform sport-related tasks is more intrinsically determined. Intrinsically motivated martial arts athletes are then more likely to practice self-regulation via deliberate and consistent use of various strategies of self-management that would promote achievement of performance goals leading to positive psychological outcomes. In contrast, martial arts players who feel that they are not part of the team, often gets negative feedback from peers and coaches, and have no choice in the decision-making, their motivation to perform the behavior tends to be less self-determined (externally regulated) and concurrently impair their self-regulation capacity resulting to negative consequences such as decrease in self-satisfaction, self-confidence and commitment. Therefore, a relationship exists between self-management and psychological outcomes. That is, the use of self-management may facilitate athletes’ psychological responses and it may function to regulate different dimensions (i.e., psychosocial, physical, and tactical) of athletes’ sports participation, thus influencing not only their behaviors but also their cognitive states.

For the last few decades, various researchers have focused on exploring both traditional and new forms of martial arts (e.g., Green and Svinth, 2010) examining athletes’ employment of self-management and its associated outcomes. While martial arts can be described in several ways, we define martial arts as combat or self defense techniques with the use of either bare hands/feet or weapons (bow and arrow, stick, and sword). Martial arts can be categorized in various approaches such as armed with weapon or unarmed, traditional or modern, or practice orientation (combat or spiritual). Hence, each form of martial arts may fall into several categories depending on the established criteria such as: armed with weapon (encing-foil, épée, and saber swords, archery (bow and arrow), kumdo (wooden stick) vs unarmed (judo, taekwondo, and wrestling); traditional (ssireum-Korea, taekwondo-Korea, and judo-Japan) vs modern (kumdo; fencing; archery; and mixed martial arts); or combat (archery; wrestling, boxing) vs spiritual (hapkido, aikido, and tai chi; Green and Svinth, 2010; Cynarski, 2019). In South Korea, scholars have been interested in martial arts education and its importance in a practitioners’ life and well-being (Kim J. et al., 2011; Choi, 2017). Further, due to the rise of competitive martial arts, researchers have shown increased interest in examining this topic such as concept of self-management of Korean athletes from various martial arts for they achieved many sporting successes in various international competitions particularly in the Olympic Games such as archery, taekwondo, judo, wrestling (Greco-Roman and freestyle), fencing, and boxing. These martial arts sports have made consistent and significant contributions to South Korea’s Olympic dominance accounting for 63% of the total medals won in the Summer Olympic Games (Olympic Games, 2021). For instance, Choi et al. (2009) reported that a high level of self-control is an essential psychological characteristic for taekwondo practitioners to achieve higher skill levels in techniques related to the martial arts and higher self-confidence. Similarly, competitive players in ssireum, judo, and wrestling with high levels of self-confidence, performance, and task orientation were found to frequently employ different self-management strategies (Han, 2008; Kim and Chun, 2010; Kim et al., 2015). Lim et al. (2015) found taekwondo athletes who practiced self-management positively influenced their sport commitment and exercise satisfaction.

Furthermore, qualitative studies provide evidences about the effectiveness of self-management in improving sport performance of athletes. Park (2000) described that training hard, yelling, singing, listening to music, and ignoring the actual date of competition were some thought- and emotional-regulating strategies employed by a judo athlete who won a gold medal in the Olympics. Lim and O’Sullivan (2016) did a case study about the effect of a systematic mental skills program on the psychological state and performance of a taekwondo Olympian and found positive changes in the athlete’s psycho-emotional condition and behaviors. Increased physical effort, positive mentality, self-confidence, decreased negative thoughts, anxiety level, and perceived stress, and improved competition performance style were observed after employing several strategies to regulate the player’s feelings, thoughts and actions. They reported that a systematic mental skills program helped the player how to better control the stressors of competition that subsequently facilitated the taekwondo athlete’s gold medal success in the Olympic Games.

Based on the results, the authors of these cited studies concluded that self-management may play a determinant role in practitioners’ psychological characteristics. Specifically, the self-management of martial arts practitioners might be the main influential factor of their exercise self-confidence, satisfaction, and commitment (Huh, 2003; Han, 2008; Chun, 2011; Kim, 2015; Kim and Cho, 2017; Kim and Kim, 2018). Here, we define satisfaction as cognitive evaluation of one’s athletic participation (Riemer and Chelladurai, 1998) while commitment is a person’s motivational desire and determination to continue sports participation (Scanlan et al., 1993).

Notwithstanding, despite the multitude of studies verifying the relationships between athletes’ self-management and exercise self-confidence, satisfaction, and commitment, no attempt has been made to systematically investigate these variables and consolidate the findings in the literature through a meta-analytic approach. We believe that a meta-analysis on the relationships between self-management and these psychological characteristics in the sports field could serve to bridge theoretical knowledge and practical implications; specifically, the provision of systematic data can help coaches/sports psychologists to identify/develop effective training interventions targeted at the development of athletes’ self-management skills—thereby potentially helping enhance athletes’ psychological well-being. Such a study could also serve to provide additional knowledge on the self-management phenomenon, thus enriching interdisciplinary literature on the topic in general and South Korea in particular. Likewise, a greater understanding of this phenomenon—and its benefits—can help athletes with no access to professional sports/mental coaches by providing information on specific aspects of their training that they can directly modify; this could potentially help them achieve an optimal psychological state, which could, in turn, allow for longer sports participation and higher performance.

Accordingly, this study aimed to meta-analyze studies on the relationships between athletes’ self-management and exercise self-confidence, satisfaction, and commitment. The following are the research questions of this study: (1) What are the overall effect sizes (ES) of the relationships among self-management and the outcome variables (i.e., exercise self-confidence, satisfaction, and commitment)? (2) What are the ES of different dimensions of self-management on the outcome variables? (3) What are the ES of the relationships among self-management and the outcome variables in different martial arts sports? and (4) What are the ES of the relationships among self-management and the outcome variables in different levels of sports participation?

## Materials and Methods

### Procedure

In the late twentieth century, narrative reviews and content analyses were popular when researchers aimed to synthesize knowledge on heterogeneous findings on certain issues, constructs/variables, and relationships (Higgins and Thompson, 2002; Kim and Cruz, 2016; Grewal et al., 2018). Compared with these two which considered to be more subjective method, meta-analyses are known to be a more rigorous alternative for synthesizing the robustness of findings in any scholarly field; namely, the meta-analytic approach has helped scholars to satisfy the needs for knowledge accumulation and development in many field (Borenstein et al., 2009; Palmatier et al., 2017; Grewal et al., 2018). Additionally, this sophisticated method allows researchers to empirically compare and combine findings across studies on specific issues and research domains.

Despite this design being valid to systematically identify empirical findings, it also presents limitations; specifically, it has some issues regarding its extracting and coding procedures and the judgements on the relevance of studies. Thus, to ensure that we found relevant and reliable answers to the research questions of the current study, we strictly followed the guidelines for meta-analyses set forth (Hunter and Schmidt, 2004; Cohen et al., 2007; Borenstein et al., 2009).

We selected electronic databases (i.e., Google Scholar, SPORTDiscus, National Library of Korea, National Digital Science Library, PubMed, PsycINFO, KERIS, National Assembly Library) to search for scholarly publications and documents. We used the following keywords: self-management; self-confidence; commitment; satisfaction; and martial arts. These were used together with the following words: sports; exercise; traditional; modern; psychological; and performance.

**FIGURE 1:**
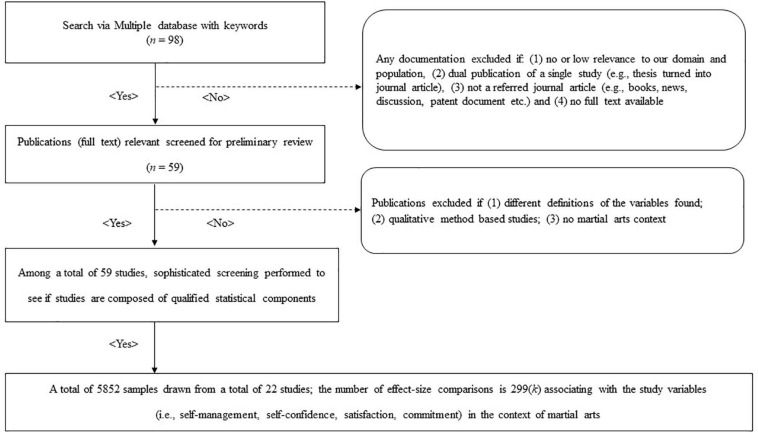
Flowchart of the study selection process.

We used the following exclusion criteria to exclude studies in the preliminary search: (1) no or low relevance to our outcome variables and population; (2) dual publication of a single study (e.g., unpublished thesis or dissertation concomitantly published as journal articles); (3) not a referred journal article (e.g., case reports, narrative review, conference proceedings, and abstracts); and (4) no full-text availability (see Figure 1).

Through this preliminary search and review process, we found a total of 59 studies. To determine their eligibility, we used the following inclusion criteria: (1) theoretical relevance (e.g., meanings of operational definitions); (2) population similarity (i.e., sample description); (3) completeness and adequacy of statistical reports; and (4) acceptance status of the Official Korean Citation Index approved by the Korea National Research Foundation. In total, 22 articles—totalizing 5852 participants—were included in this meta-analysis (see Table 1). From the included articles, the featured martial arts were boxing, judo, fencing, wrestling, archery, taekwondo, kumdo, and ssireum. Taekwondo, ssireum and kumdo are martial arts developed in Korea. Ssireum is a traditional martial art wherein players wear a cloth-sash (satba) around their waist and right thigh, and opponents lock on to each other’s satbas. In order to win, ssireum players have to throw their opponents to the sandy ground using their strength and various skills (Noh et al., 2015). Kumdo is a modern martial art adopted from Japanese kendo that uses a bamboo sword to strike opponents (Green and Svinth, 2010). Taekwondo is the representative martial art of Korea with emphasis on kicking techniques targeting the body and head. It evolved as a competitive sport and has been part of the Olympic Games since its debut in 1988 (Green and Svinth, 2010; Olympics, 2021d). Boxing and wrestling are combat unarmed sports included in both ancient and modern Olympic games. The former uses striking skills using the hands, while the latter focuses on grappling and pinning movements to attack opponents (Olympics, 2021b,e). Judo is a martial art originated from Japan and an official event in the Olympics. It mostly comprises of throwing techniques to subdue rivals (Green and Svinth, 2010; Olympics, 2021c). Fencing is a sword fighting style of martial art originated in Europe (Windsor, 2018) whereas archery is originally a form of combative martial art used for hunting and war before it became a competitive sport during the middle ages (Green, 2001). As a modern Olympic sport, the archer uses a weapon of bow and arrows to shoot a 122 cm diameter target 70 m away (Tokyo 2020, 2020; Olympics, 2021a).

**TABLE 1:** Summary of Korean self-management studies in martial arts.

#	References	*n*	Martial arts type	Participants	DV
1	Chun, 2011	221	Judo	MH recreational participant	Satisfaction and commitment
2	Lim et al., 2015	281	Taekwondo	MH recreational participant	Satisfaction and commitment
3	Kim J. P. et al., 2011	150	Boxing	MH recreational participant	Satisfaction
4	Lim et al., 2010	255	Taekwondo	Professional	Satisfaction
5	Kim and Kwon, 2018	269	Taekwondo	Collegiate athlete	Satisfaction
6	Seol and Choi, 2019	275	Taekwondo	MH elite athlete	Satisfaction
7	Lee and Nam, 2018	295	Taekwondo	Collegiate athlete	Satisfaction and commitment
8	Park and Shin, 2015	323	Kumdo	Collegiate athlete	Satisfaction
9	Park et al., 2018	300	Kumdo	Collegiate athlete	Satisfaction
10	Moon and Park, 2008	199	Archery	MH elite, collegiate and professional	Self-confidence
11	Song and Lee, 2015	231	Taekwondo	Collegiate athlete	Satisfaction and self-confidence
12	Kim, 2017	297	Taekwondo	Collegiate athlete	Commitment
13	Kim, 2010.	235	Judo	Collegiate athlete	Self-confidence
14	Kim et al., 2017	218	Fencing	Collgiate and professional	Commitment
15	Kong, 2017	339	Ssireum	MH elite athlete	Commitment
16	Kim and Cho, 2017	228	Mudo	Collegiate athlete	Self-confidence
17	Hur et al., 2011	147	Ssireum	Collegiate athlete	Self-confidence
18	Kim, 2015	206	Taekwondo	Collegiate athlete	Self-confidence
19	Kim and Kim, 2018	276	Wrestling	Collegiate and professional	Commitment
20	Han, 2008	409	Wrestling	MH elite and professional	Self-confidence
21	Kim and Chun, 2010	360	Ssireum	MH elite athlete	Self-confidence
22	Kim et al., 2015	338	Judo	MH elite, collegiate and professional	Self-confidence

*MH, mid-high school athletes; *n*, sample size; and DV, dependent variable.*

### Data Analysis

To compute ES based on the correlation of the study variables, we converted the sample size (*n*) and correlation coefficient values *I* of each study into Fisher’s *z*-scores; this was performed based on the meta-analytic procedure outlined in previous research (Higgins and Thompson, 2002; Borenstein et al., 2009; Kim and Cruz, 2016). Furthermore, we considered the following as moderator variables: level of sports participation [i.e., middle/high school student athletes and recreational participants (MHR); and collegiate student and professional athletes (CEPA)], and type of martial arts sport. Owing to the lack of studies in non-elite sports practitioners, we chose to combine the high school and recreational players into one group, while the collegiate and professional athletes into another group.

To accurately estimate the ES from the selected studies, we used the Comprehensive Meta-Analysis version II program. Since the target population has unique and specific characteristics, we followed Borenstein et al. (2009)’s recommendations, thereby ensuring that the basic assumption of accepting the random effect model (i.e., a single population assumption) was adequately met. We assessed publication bias using funnel plots. The plot results were distributed horizontally, which signified rejection of publication bias. We followed Borenstein et al. (2009)’s and Cohen et al. (2007)’s recommendations on the interpretation of ES levels, which are the following: an ES of 0.10 equals to a small; an ES of 0.25 equals to a medium; and an ES of 0.40 to a large heterogeneity.

By estimating the *I*^2^ statistic (*I*^2^ = 100% × (*Q* – *df*)/Q; where *Q* is the Cochran’s heterogeneity statistic), we assessed the homogeneity of all relationships between the outcome variables. Based on Higgins et al. (2003), the *I*^2^ index values of 25, 50, and higher than 75% were interpreted as small, moderate, and large levels of heterogeneity of individual effects, respectively. We conducted the heterogeneity assessment of all accepted studies because, as remarked by Hunter and Schmidt (2004), this is one of the essential procedures of a meta-analysis.

## Results

### What Are the Overall Effect Sizes of the Relationships Between the Outcome Variables (i.e., Exercise Self-Management, Self-Confidence, Satisfaction, and Commitment)?

Table 2 presents a summary of the overall ES for the relationships between self-management and exercise self-confidence, satisfaction, and commitment. The results for the overall ES of self-management showed positive moderate effect on athletic satisfaction (ES = 0.329) and self-confidence (ES = 0.359) and positive large effect on exercise commitment (ES = 0.407).

**TABLE 2:** Overall effect size levels of self-management on psychological outcomes.

Variable	*n*	ES	-95%CI	+95%CI	*p*	*Q*	*I*^2^	Tau
Athletic satisfaction	83	0.329	0.288	0.369	0.000	971.746	91.561	0.2035
Exercise commitment	52	0.407	0.375	0.439	0.000	268.411	80.999	0.128
Self-confidence	164	0.359	0.346	0.371	0.000	378.335	56.9165	0.0071

**n*, Number of selected studies; ES, Effect Size; and CI, Confidence interval.*

### What Are the ES of Different Dimensions of Self-Management on Exercise Self-Confidence, Satisfaction, and Commitment?

Table 3 presents the ES for the effect of the dimensions of self-management on the outcome variables. Results showed that all four dimensions of self-management had the highest ES for exercise commitment (CIs ranged from 0.357 to 0.445), followed by exercise self-confidence (CIs ranged from 0.325 to 0.378) and satisfaction (CIs ranged from 0.237 to 0.375). Moreover, the effects of all dimensions of self-management on the outcome variables were generally moderate except the mental and training dimensions on exercise commitment that yielded positive large effects.

**TABLE 3:** Effects of aspects of self-management on psychological outcomes.

Aspects (SM)	Variables	*k*	ES	−95%CI	+95%CI	*Q*	*I*^2^	Tau
Interpersonal	Athletic satisfaction	21	0.358	0.263	0.447	292.3	93.500	0.234
	Exercise commitment	13	0.391	0.343	0.438	32.74	63.347	0.081
	Self-confidence	41	0.376	0.353	0.400	80.90	50.558	6.32E
Physical	Athletic satisfaction	21	0.237	0.160	0.311	180.74	88.935	0.175
	Exercise commitment	13	0.357	0.291	0.419	57.42	79.102	0.120
	Self confidence	41	0.325	0.302	0.347	68.56	41.66	5.28E
Mental	Athletic satisfaction	21	0.345	0.267	0.418	210.73	90.509	0.191
	Exercise commitment	13	0.434	0.368	0.496	65.524	81.686	0.1307
	Self confidence	41	0.378	0.359	0396	50.891	21.401	0.032E-02
Training	Athletic satisfaction	21	0.375	0.301	0.446	205.138	90.250	0.188
	Exercise commitment	13	0.445	0.369	0.514	86.559	86.137	0.154
	Self confidence	41	0.359	0.325	0.392	154.498	74.109	0.106

**k*, Number of comparisons; CI, Confidence interval; and SM, Self-Management.*

### What Are the ES of the Relationships Between the Outcome Variables (i.e., Self-Management and Exercise Self-Confidence, Satisfaction, and Commitment) in Different Martial Arts Sports?

Table 4 presents the ES for the effect of self-management on the outcome variables by type of martial arts (i.e., as a moderator). Results showed that the effect of self-management on exercise satisfaction in judo was larger than all other martial arts; in Kumdo, this effect was also significant, albeit relatively lower. The effect of self-management on exercise commitment in Ssireum was the largest, followed by boxing, and judo. The effect of self-management on exercise self-confidence showed similar levels across different martial arts sports (CIs ranged from 0.328 to 0.416), and it was the largest in wrestling (95% CIs = 0.39 to 0.45, ES = 0.416, and *p* = 0.05).

**TABLE 4:** Effects of self-management on psychological outcomes based on different martial arts.

Variables	TM	*k*	ES	−95%CI	+95%CI	*Q*
Athletic satisfaction	Boxing	8	0.309	0.157	0.446	73.675
	Judo	12	0.413	0.337	0.483	58.448
	Kumdo	24	0.284	0.219	0.347	215.127
	TKD	39	0.335	0.265	0.400	580.009
Exercise commitment	Boxing	4	0.448	0.307	0.569	12.455
	Fencing	8	0.388	0.301	0.469	31.121
	Judo	4	0.419	0.309	0.518	10.954
	Ssirem	4	0.582	0.537	0.624	4.589
	TKD	24	0.370	0.334	0.404	69.208
	Wrestling	8	0.410	0.313	0.499	50.337
Self confidence	Archery	16	0.395	0.351	0.436	31.090
	Fencing	4	0.364	0.290	0.434	4.691
	Ssirem	36	0.335	0.317	0.353	32.797
	Judo	32	0.342	0.317	0.367	58.147
	TKD	40	0.328	0.297	0.358	99.967
	Wrestling	20	0.416	0.385	0.446	51.752

**k*, Number of comparisons; CI, Confidence interval; TM, Type of martial arts; and TKD, Taekwondo.*

### What Are the ES of the Relationships Between the Outcome Variables (i.e., Self-Management and Exercise Self-Confidence, Satisfaction, and Commitment) in Different Levels of Sports Participation?

Table 5 shows the differences in ES levels by level of sports participation. Results showed that the effect of self-management was greater in the MHR group (CIs ranged from 0.362 to 0.448) than the CEPA group on all outcome variables.

**TABLE 5:** Effects of self-management on psychological outcomes based on level of sports participation.

Variables	MIL	*n*	ES	−95%CI	+95%CI	*Q*	*I*^2^	Tau
Athletic satisfaction	MHR	36	0.362	0.305	0.416	325.784	89.256	0.183
	CEPA	47	0.304	0.246	0.361	621.054	92.593	0.213
Exercise commitment	MHR	20	0.448	0.394	0.499	105.806	82.042	0.135
	CEPA	32	0.380	0.342	0.418	135.853	77.181	0.112
Self confidence	MHR	68	0.369	0.353	0.386	141.042	52.496	5.83E-02
	CEPA	96	0.349	0.331	0.369	232.047	59.060	8.32E

*MIL, Martial arts involvement level; MHR, Middle and high School athlete and recreational participants; and CEPA, Collegiate elite and professional athletes.*

## Discussion

This study conducted a meta-analysis to examine the overall ES of the relationships between self-management and exercise self-confidence, satisfaction, and commitment of martial arts practitioners in Korea. We also examined the influence of each dimension of self-management on the outcome variables, and the effects of self-management on the outcome variables in different martial arts sports and in different levels of sports participation.

Overall, the results showed moderate to strong effects between self-management and exercise self-confidence, satisfaction, and commitment. Specifically, exercise commitment was shown to be greatly influenced by self-management, whereas exercise self-confidence and satisfaction were only be moderately influenced; namely, martial art practitioners who practiced self-management (i.e., on the physical, interpersonal, mental, and training dimensions of their athletic career) not only felt higher exercise satisfaction and self-confidence but also higher exercise commitment. Coaches, sports practitioners, and sports psychologists should, hence, implement self-management training not only to athletes who want to sustain or enhance their positive mental state but also to those who tend to lack exercise self-confidence and satisfaction, and even more so to those who display diminished commitment in their sports participation.

### The Influence of Different Dimensions of Self-Management on Exercise Self-Confidence, Satisfaction, and Commitment

By analyzing the contributions of each dimension of self-management on the three psychological outcome variables, we observed that the interpersonal dimension had a moderately positive impact on exercise commitment, followed by exercise self-confidence and satisfaction. Namely, martial arts practitioners who employed interpersonal self-management (i.e., managed their interactions/communication with their teammates and coaches) moderately enhanced their commitment, their beliefs in the ability to successfully perform a sports-related behavior, and their satisfaction. Hence, to promote exercise commitment, self-confidence, and satisfaction, athletes should be proficient at directly regulating their interpersonal relationships with teammates and coaches; this can be done by having greater awareness over the need to treat those surrounding the athlete nicely and to display behaviors that are expected/desired by each team member, coach, or the group. Especially, coaches and teammates who are casted in the roles of leadership positions should demonstrate support for an acceptance and mindfulness-oriented intervention during the practice circumstance.

The physical dimension of self-management had a moderately positive effect on exercise commitment and self-confidence, but only a small positive effect on exercise satisfaction. Namely, martial arts practitioners who self-monitored the physical aspect of their training had higher exercise commitment and self-confidence, but not high satisfaction with the exercise. The small effect found in the relationship between physical self-management and exercise satisfaction may be attributed to the goal of sports participation—of winning. We deem feasible to think that many athletes do not perceive the self-management of their sleep regimen and eating patterns as an essential part of a successful sports performance, and this may explain the aforementioned small effect. Another reason might be the leadership style of the coach and its consequence on satisfaction (Chelladurai, 1993; Kim and Cruz, 2016). Specifically, Kim and Cruz (2016) conducted a meta-analysis on the relationship between leadership behavior and exercise/athletic satisfaction, finding that an autocratic style contributed to a small positive effect of leadership behavior on athletic satisfaction. In Korea, athletes’ daily activities and training schedules are generally controlled by the coach; accordingly, the autocratic style of coaches might have prevented athletes from fully controlling the physical dimension of their training by themselves, thereby leading to diminished satisfaction with their sports participation. Hence, coaches may need to provide an autonomy-supportive environment to their players, namely, give them freedom to control even their activities outside training; these may lead to higher levels of satisfaction in athletes.

The mental dimension of self-management had a large positive effect on exercise commitment and moderate positive effects on exercise self-confidence and satisfaction. Namely, martial art practitioners who could directly manage their negative thoughts, stress, and anxiety had moderate levels of satisfaction and self-confidence, while concomitantly reporting considerably high levels of exercise commitment. It is possible that participants in this study used self-management strategies such as self-talk and imagery to manage their mental concerns, and these may have led not only to increased self-confidence and satisfaction but also increased commitment. This idea corroborates that found in Hatzigeorgiadis et al. (2011); they conducted a meta-analytic review on the effects of self-talk on task performance, reporting that self-talk (i.e., a self-instructional strategy) was effective in enhancing learning and sports performance owing to its various functions in people’s mental state (Theodorakis et al., 2008). Similarly, motivational general-arousal imagery was shown to decrease athletes’ anxiety (Strachan and Munroe-Chandler, 2006), while motivational general-mastery imagery increased their self-confidence (Callow and Hardy, 2001; Callow et al., 2001). Nonetheless, these two latter studies only partially corroborate our findings because the magnitude of the relationship between the mental dimension of self-management was large for exercise commitment and moderate for self-confidence and satisfaction. Therefore, martial arts players who want more persistence in their sports participation, better trust in their own skills and abilities, and higher sense of sports fulfillment should consider practicing positive self-talk rather than negative self-talk and learning imagery training to alleviate their cognitive concerns prior to, during, and even after sports training and competition.

The training dimension of self-management showed a moderate effect on exercise satisfaction and self-confidence and a large effect on exercise commitment. Namely, martial arts practitioners who were capable of developing and organizing their own practice sessions for physical and skill improvements, and to self-assess their training efforts and performance, demonstrated increased exercise satisfaction, self-confidence, and even greater exercise commitment. The large influence of the training dimension of self-management on exercise commitment might be explained by study participants’ personality; they might have had high levels of self-confidence. Self-confidence was found to affect how people choose and chase their goals, promoting positive emotions, enhancing concentration, and increasing persistence (Theodorakis, 1995; Bandura, 1997; Weinberg and Gould, 2015). Thus, martial arts practitioners, who had high self-confidence about self-management may have used appropriate self-management skills to help them focus on achieving their target training and competition goals, thereby resulting in higher levels of commitment (Kim, 2010; Hur et al., 2011; Jeon, 2016; Kong, 2017). Moreover, participants included in the current study were mostly engaged in competitive sports, which denote that they aspire to achieve valuable opportunities and to become successful. Previous studies have shown the effect of athletes’ subjective outcomes on intrinsic motivation. When athletes focus on the performance outcomes of competition rather than actual result, they tend to feel more intrinsically motivated (Weinberg and Gould, 2015) which can directly affects their commitment (Scanlan et al., 1993; Weiss and Aloe, 2019).

Summarizing, all dimensions of self-management showed a moderate effect on exercise self-confidence. Regarding the relationship between self-management and exercise commitment, the mental and training dimensions of self-management had a large contribution, while the interpersonal and physical dimensions had a moderate contribution. Regarding the relationship between self-management and exercise satisfaction, the interpersonal, mental, and training dimensions showed a moderate contribution, while the physical showed a small contribution.

### Self-Management and Outcome Variables in Different Martial Arts Sports

The influence of self-management on the outcome variables ranged from moderate to large ES across different martial arts sports. Specifically, self-management had large effects on exercise satisfaction in judo, exercise self-confidence in wrestling, and exercise commitment in Ssireum, boxing, judo, and wrestling. This result might be explained by players’ achievement orientation, level of experience, and level of success. Previous studies showed that successful and highly experienced wrestlers and judo players were mostly driven toward achieving task-oriented goals such as personal mastery and higher competence attainment (Han, 2008; Park, 2018). Likewise, successful Ssireum players generally focused on regulating their cognitive and emotional states to build their physical and mental competence (Kim and Chun, 2010; Phillips, 2011; Meyer and Bittman, 2018). These personal factors of athletes could have had positively affected their use of self-management strategies and consequently led to substantially higher exercise satisfaction, self-confidence, and commitment. The findings suggest that the positive effects of self-management on psychological outcomes may be moderated by individuals’ characteristics and confirms the notion that successfully achieving goals while employing self-management could also enhance self-confidence (Dishman et al., 2005). Meanwhile, athletes’ autonomy-supportive coaches may also explain these positive results. In an in-depth interview with Korean Olympic archers, several players stated that when their coach supported the goals they decided to pursue and gave them permission to express their views about the training schedule, they felt more dedicated and accountable to achieve their set goals (Kim and Park, 2020). This finding corroborates our results, demonstrating that commitment can significantly and positively be affected by athletes’ control over their training environment and interpersonal communication with their coach. Still, we remark that future studies should examine the specific self-management strategies (e.g., coping skills, mental imagery, etc.) that these practitioners use to regulate their thoughts, emotions, and experiences in various dimensions of their sports participation. This would help pave the pathway for well-informed decision-making regarding interventions to enhance martial arts practitioners’ exercise commitment, self-confidence, and satisfaction.

### Self-Management and Outcome Variables Based on Level of Sports Participation

Our results showed that the effects of self-management on exercise satisfaction, commitment, and self-confidence were moderate to large in both groups; still, the ES were higher in the MHR than in CEPA. This means that MHR martial arts practitioners who were capable of managing their relationships with others, control their emotions, and plan their training goals to achieve athletic success reported higher perceived satisfaction, confidence, and exercise commitment. The diminished influence of self-management in CEPA (i.e., elite practitioners) may be explained by external factors that might inhibited their capacity to practice self-management, thereby lowering their levels of exercise satisfaction, commitment, and self-confidence.

Elite or professional (CEPA) level competition is a sporting environment that provokes tremendous amount of physiological and psychological stresses for players (Jeon, 2016). These stressors can be winning expectation and injury. For example, Taekwondo is considered one of the most injury prone martial arts sport. In elite players, the injury incidence rate ranged from 21 to 140 per 1000 athlete exposures overall, whereas 79.0 per 1000 athlete exposures during competition were recorded (Son et al., 2020). Moreover, Taekwondo has been in the top 5 sports with highest rate of injury prevalence in the Olympics since 2008 (Son et al., 2020). It is therefore vital for players to be cognizant of ways to avoid injuries and even cope when injury occurs. Without the ability to manage unpleasant responses concerning injury-related stressors, athletes’ perception of competence, satisfaction and commitment are likely to diminish. This notion coincides with previous research in taekwondo elite athletes (Im et al., 2015) in which interpersonal, mental, training, and physical aspects of self-management were found to be significant predictors of exercise satisfaction and commitment. In contrast, lower level competition players may have had focused on skill mastery and personal satisfaction to learn new things as their main objectives in sport participation. While participating, high school and recreational players (MHR) might had felt positive social support from peers and coaches that led to their higher interest, control and commitment to endure the challenges of training and competition as well as sport fulfillment. This reason coincides with Bandura’s (1997) self-efficacy and Deci and Ryan’s (1985) self-determination theories explaining how self-efficacy and the kind of motivation regulation may affect one’s psychological outcomes.

The results highlights how self-regulation of thoughts, feelings, and behaviors to affect performance outcomes is dependent on athletes’ level of competition. More so, it appears that as level of competition becomes more competitive or more focused on the objective outcomes rather than performance outcomes, higher level athletes’ capacity to self-regulate aspects of their sports participation during training and competition is likely to be more challenging than lower level players owing to a more stressful sport environment. Hence, we argue that a negative association exist between these two variables and therefore warrants further investigation. Nonetheless, to further enhance Korean practitioners’ exercise self-confidence, satisfaction, and commitment, stakeholders (coaches, sport psychologists, team physicians, and athletic trainers) should devise educational interventions that underscore the importance of self-management in sports. For instance, since the training and mental dimensions of self-management were found to greatly increase practitioners’ exercise satisfaction, commitment, and self-confidence, stakeholders could devise interventions to teach athletes, particularly elite players (e.g., Kumdo and Taekwondo, which showed the lowest ES) how to independently plan training goals that are specific, achievable, and relevant to their needs. This can be overlearning sets of taekwondo kicking combinations during practice which may help in stronger memory retention and automatic processing for the learned motor skills (Schmidt and Lee, 2011) that can be positively transferred in competitive setting. Likewise, taekwondo players can use self-management strategies like positive self-talk, breathing techniques or meditation to regulate their arousal level and physical pain during training and up to the time prior to a match.

### Limitations and Future Directions

This meta-analysis study has potential limitations. Even though this study provides valuable input on understanding psychological influence of self-management on those underlying variables, the studies selected for this meta-analysis were mainly Korean articles and the results may therefore not be applicable to martial arts athletes/practitioners outside of this setting. However, the practice of martial arts is part of Korea’s rich culture and history and understanding its philosophical values and cultivation in Korean people’s lives is therefore a worthy endeavor. More so, South Korea is one of the few countries in the world where higher education institutions have fully-operating martial arts-related departments and colleges. These departments and colleges underpin that understanding the various reasons as to why groups of people embrace martial arts in their lives is one of the core objectives of academic scholars and practitioners of martial arts. Lastly, as some martial arts evolved and became competitive sports, a new set of South Korean martial arts participants (competitive athletes) emerged and insights about their involvement such as the use of self-management and its corresponding psychological outcomes warrant attention. We believe that these reasons underscore the importance and uniqueness of the current meta-analysis study. For future research, more studies from other continents or countries and other type of sports are warranted to gain a better insight into self-management in martial arts athletes and its effects on exercise self-confidence, satisfaction, and commitment. Although martial arts tend to have opponents in a competitive setting, martial arts without opponents (archery, martial arts as demonstration sport) can be another topic to consider particularly how various psychological variables can be affected based on the type of self-management employed.

The ES estimations for the moderator analyses can be another limitation because we had a small number of samples; unfortunately, it is common to observe larger effects when the samples are small. In the current study, particularly when analyzing different martial arts sports as a moderator, some sports with a small number of comparisons (*k* = 4; e.g., boxing, fencing) yielded large ES. Hence, results from these sports should be evaluated with caution, and studies that examine athletes’ cognitive responses toward the use of self-management on these sports are warranted.

## Conclusion

Overall, this meta-analysis showed the following: the effectiveness of self-management for enhancing exercise self-confidence, satisfaction, and commitment in Korean martial arts practitioners; the moderate to large contributions among different dimensions of self-management on the outcome variables—particularly exercise commitment; and how different martial arts sports and level of sports participation moderate the relationships between self-management and exercise self-confidence, satisfaction, and commitment. These results further advance our knowledge on self-management and underscore the importance of self-management interventions in sports. They also extend our knowledge concerning martial arts, particularly regarding martial arts practice of Korean participants.

To our knowledge, this was the first study to systematically consolidate, review, and analyze—using a more rigorous quantitative method—relevant studies on self-management and the three analyzed cognitive outcomes. Summarizing, for athletes to show higher self-confidence in—and commitment and satisfaction with—their sports, coaches, sports psychologists, and sports practitioners should incorporate the concept of self-management in their respective sports practice.

## Data Availability Statement

The raw data supporting the conclusions of this article will be made available by the authors, without undue reservation.

## Author Contributions

H-DK and AC conceptualized the research project and contributed to the writing of the manuscript (from the initial draft to the final manuscript). H-DK analyzed the data. Both authors contributed to the article and approved the submitted version.

## Conflict of Interest

The authors declare that the research was conducted in the absence of any commercial or financial relationships that could be construed as a potential conflict of interest.

## References

[B1] Arnold R. Wagstaff C. R. Steadman L. Pratt Y. (2017). The organisational stressors encountered by athletes with a disability. *J. Sports Sci*. 35 1187–1196. 10.1080/02640414.2016.1214285 27472292

[B2] Bandura A. (1977). Self-efficacy: toward a unifying theory of behavioral change. *Psychol. Rev*. 84 191–215. 10.1037//0033-295x.84.2.191 847061

[B3] Bandura A. (1997). *Self-Efficacy: The Exercise of Control.* New York, NY: Freeman.

[B4] Borenstein M. Hedges L. V. Higgins J. P. T. Rothstein H. R. (2009). *Introduction to Meta-Analysis.* Hoboken, NY: Wiley.

[B5] Callow N. Hardy L. (2001). Types of imagery associated with sport confidence in netball players of varying skill levels. *J. Appl. Sport Psychol.* 13 1–17. 10.1080/10413200109339001

[B6] Callow N. Hardy L. Hall C. (2001). The effects of a motivational general-mastery imagery intervention on the sport confidence of high-level badminton players. *Res. Q Exerc. Sport.* 72 389–400. 10.1080/02701367.2001.10608975 11770788

[B7] Chelladurai P. (1993). “Leadership,” in *Handbook of Research on Sport Psychology*, eds Singer R. N. Murphey M. Tennant L. K. (New York, NY: MacMillan), 647–671.

[B8] Choi J. Ko G. Lim T. (2009). The change of self-regulation according to Taekwondo discipline. *Injoma.* 11 181–196. 10.35277/kama.2009.11.3.181

[B9] Choi S. K. (2017). Analysis and suggestions of Korean junior high school martial arts curriculum. *Int. J. Martial Arts* 2 25–29. 10.22471/martialarts.2017.2.2.25

[B10] Chun (2011). The influence of member satisfaction or exercise commitment by Judo players’ self-management on instruction effectiveness. *Injoma* 13 59–76.

[B11] Cintineo H. P. Arent S. M. (2019). Anticipatory salivary cortisol and state anxiety before competition predict match outcome in division I collegiate wrestlers. *J. Strength Cond. Res.* 33 2905–2908. 10.1519/JSC.0000000000003376 31490432

[B12] Cohen L. Manion L. Morrison K. (2007). *Research Methods in Education*, 6th Edn. Abingdon: Routledge.

[B13] Collins J. Durand-Bush N. (2014). Strategies used by an elite curling coach to nurture athletes’ self-regulation: a single case study. *J. Appl. Sport Psychol.* 26 211–224. 10.1080/10413200.2013.819823

[B14] Critchfield T. S. Vargas E. A. (1991). Self-recording, instructions, and public self-graphing: effects on swimming in the absence of coach verbal interaction. *Behav. Modif.* 15 95–112. 10.1177/01454455910151006

[B15] Cynarski W. J. (2019). *Martial Arts and Combat Sports. Towards the General Theory of Fighting Arts.* Gdańsk: Wydawnictwo Naukowe Katedra.

[B16] Deci E. L. Ryan R. M. (1985). *Intrinsic Motivation and Self-Determination in Human Behavior.* New York, NY: Plenum Press.

[B100] Deci E. L. Ryan R. M. (2000). The “what” and “why” of goal pursuits: human needs and the self-determination of behavior. *Psychol. Inq.* 11, 227–268. 10.1207/S15327965PLI1104_01

[B17] Dishman R. K. Motl R. W. Sallis J. F. Dunn A. L. Birnbaum A. S. Welk G. J. (2005). Self-management strategies mediate self-efficacy and physical activity. *Am. J. Prev. Med.* 29 10–18. 10.1016/j.amepre.2005.03.012 15958246 PMC2435261

[B18] Evans L. Wadey R. Hanton S. Mitchell I. (2012). Stressors experienced by injured athletes. *J. Sports Sci.* 30 917–927. 10.1080/02640414.2012.682078 22551525

[B19] Fletcher D. Sarkar M. (2012). A grounded theory of psychological resilience in Olympic champions. *Psychol. Sport Exerc.* 13 669–678. 10.1016/j.psychsport.2012.04.007

[B20] Furlonger B. E. Oey A. Moore D. W. Busacca M. Scott D. (2017). Improving amateur indoor rock climbing performance using a changing criterion design within a self-management program. *Sport J.* 19 1–16.

[B21] Gerhardt M. W. (2007). Teaching self-management: the design and implementation of self-management tutorials. *J. Educ. Bus.* 83 11–18. 10.3200/JOEB.83.1.11-18

[B22] Grady P. A. Gough L. L. (2014). Self-management: a comprehensive approach to management of chronic conditions. *Am. J. Public Health.* 104 e25–e31. 10.2105/AJPH.2014.302041 24922170 PMC4103232

[B23] Green T. A. (2001). *Martial Arts of the World. An Encyclopedia Volume one: A-Q.* Santa Barbara, CA: ABC-CLIO, LLC.

[B24] Green T. A. Svinth J. R. (2010). *Martial Arts of the World: an Encyclopedia of History and Innovation.* Santa Barbara, CA: ABC-CLIO, LLC.

[B25] Grewal D. Puccinelli N. Monroe K. B. (2018). Meta-analysis: integrating accumulated knowledge. *J. Acad. Mark. Sci.* 46 9–30. 10.1007/s11747-017-0570-5

[B26] Guillet E. Sarrazin P. Carpenter P. J. Trouilloud D. Cury F. (2002). Predicting persistence or withdrawal in female handballers with social exchange theory. *Int. J. Psychol*. 37 92–104. 10.1080/00207590143000243

[B27] Han T. J. (2008). The relationship among amateur wrestlers’ achievement goal orientation, self-management and sport self-confidence. *Korean J. Sport Psychol.* 19 35–52.

[B28] Hanton S. Fletcher D. Coughlan G. (2005). Stress in elite sport performers: a comparative study of competitive and organizational stressors. *J. Sports Sci.* 23 1129–1141. 10.1080/02640410500131480 16194989

[B29] Hatzigeorgiadis A. Zourbanos N. Galanis E. Theodorakis Y. (2011). Self-talk and sports performance: a meta-analysis. *Perspect. Psychol. Sci.* 6 348–356. 10.1177/1745691611413136 26167788

[B30] Higgins J. P. T. Thompson S. G. (2002). Quantifying heterogeneity in a meta-analysis. *Stat. Med.* 21 1539–1558. 10.1002/sim.1186 12111919

[B31] Higgins J. P. Thompson S. G. Deeks J. J. Alstman D. G. (2003). Measuring inconsistency in meta-analyses. *BMJ* 327, 557–560. 10.1136/bmj.327.7414.557 12958120 PMC192859

[B32] Huh J. H. (2003). Development and validation of athletes’ self-management questionnaire. *Korean Society Sport Psychol.* 14 95–109.

[B33] Hunter J. E. Schmidt F. L. (2004). *Methods of Meta-Analysis: Correcting Error and Bias in Research Findings*, 2nd Edn. Thousand Oaks, CA: Sage.

[B34] Hur Y. Shon J. H. Jung C. S. (2011). The effect of self-management on college Ssireum player’s sport-confidence. *J. Coach Dev.* 13 23–30.

[B35] Im J. H. Song S. Y. Seok R. (2015). The influence of Taekwondo athletes’ self-management on sport commitment and exercise satisfaction. *J. Korean Womens Sports Associat.* 29 177–192. 10.16915/jkapesgw.2015.06.29.2.177

[B36] Jeon K. Y. (2016). The effect of confidence and stress of college judo players on their performance. *J. Digital Converg.* 14 545–553. 10.14400/JDC.2016.14.12.545

[B37] Jung Y. Takeuchi N. (2018). A lifespan perspective for understanding career self-management and satisfaction: the role of developmental human resource practices and organizational support. *Hum. Relat.* 71 73–102. 10.1177/0018726717715075

[B38] Kim B. S. Lee S. K. Kim J. H. (2017). The effect of fencing athletes’ self-management on exercise flow and concentration. *Korean J. Sport* 15 785–794.

[B39] Kim H. D. Cruz A. B. (2016). The influence of coaches’ leadership styles on athletes’ satisfaction and team cohesion: a meta-analytic approach. *Int. J. Sports Sci. Coach.* 11 900–909. 10.1177/1747954116676117

[B40] Kim H. I. (2017). The effect of university Taekwondo players’ multidimensional perfectionism on self-management and exercise flow. *Korean J. Sport* 15 669–672.

[B41] Kim J. Dattilo J. Heo J. (2011). Taekwondo participation as serious leisure for life satisfaction and health. *J. Leis. Res.* 43 545–559. 10.1080/00222216.2011.11950249

[B42] Kim J. K. (2015). The effect of university Taekwondo players’ exercise passion on self-management and sport confidence. *Korean J. Sport Sci.* 24 281–292.

[B43] Kim J. P. Jung Y. C. Kim J. Y. (2011). Influence of boxers’ self-management upon exercise flow and exercise satisfaction. *Yongin Univ. J. Martial Arts Inst.* 22 151–162.

[B44] Kim J. W. Kim Y. E. Moon H. S. (2015). The mediating effects validation of sports stress on self-management and confidence by Judo players. *Injoma* 17 91–106. 10.35277/kama.2015.17.1.91

[B45] Kim J. Y. Kwon C. D. (2018). How self-management affects exercise satisfaction and exercise adherence for Taekwondo players? *Korean J. Sport Sci.* 27 361–371. 10.35159/kjss.2018.04.27.2.361

[B46] Kim K. S. Chun G. Y. (2010). The influence of self-management on self-confidence and athletic performance of Ssireum players. *Injoma* 12 65–80. 10.35277/kama.2010.12.2.65

[B47] Kim M. J. Cho S. L. (2017). Verification of relationship model among achievement goal orientation, self-management, and sport confidence of martial arts athletes in university. *Korean J. Sport* 15 713–724.

[B48] Kim N. I. (2010). Relationship among self-management, sport confidence, and exercise flow for collegiate Judo players. *Korean J. Sport* 8 37–47.

[B49] Kim S. K. Kim Y. N. (2018). The effect of wrestlers’ self-management on exercise flow and concentration. *J. Sport* 16 645–654.

[B50] Kim Y. Park I. (2020). “Coach really knew what I needed and understood me well as a person”: effective communication acts in coach-athlete interactions among Korean Olympic archers. *Int. J. Environ. Res. Public Health.* 17:3101. 10.3390/ijerph17093101 32365657 PMC7246789

[B51] Kong S. B. (2017). The structural relationship among adolescent Ssireum players passion, self-management, and exercise flow. *J. Sport Leisure Stud.* 67 209–219. 10.51979/kssls.2017.02.67.209

[B52] Lee Y. J. Nam M. H. (2018). The effect of Taekwondo demonstration team self-management on exercise commitment and Taekwondo demonstration satisfaction. *Korean J. Sport* 16 95–105.

[B53] Lim J. H. Song S. Y. Seok R. (2015). A study on the effect of Taekwondo athletes’ self-management on sport commitment and exercise satisfaction. *J. Korean Assoc. Phys. Educ. Sport Girls Women* 29, 177–192. 10.16915/jkapesgw.2015.06.29.2.177

[B54] Lim S. H. Song S. R. Park S. S. (2010). The structural relationship among achievement goal orientation, self-management, and athletic satisfaction of Taekwondo demonstration athlete. *Korean J. Sport Sci.* 19 357–368.

[B55] Lim T. O’Sullivan D. M. (2016). Case study of mental skills training for a taekwondo olympian. *J. Hum. Kinet.* 50 235–245. 10.1515/hukin-2015-0161 28149361 PMC5260659

[B56] Lorig K. R. Holman H. (2003). Self-management education: history, definition, outcomes, and mechanisms. *Ann. Behav. Med*. 26 1–7. 10.1207/S15324796ABM2601_01 12867348

[B57] Massey W. V. Meyer B. B. Naylor A. H. (2015). Self-regulation strategies in mixed martial arts. *J. Sport Behav.* 38 192–206.

[B58] Meyer M. J. Bittman H. (2018). Why do people train martial arts? Participation motives of German and Japanese karateka. *Societies* 8:128. 10.3390/soc8040128

[B59] Moon H. S. Park J. S. (2008). The relationship between archers’ self-management and self-confidence. *Korean J. Sport Psychol.* 19 19–32.

[B60] Noh J. W. Park B. S. Kim M. Y. Lee L. K. Yang S. M. Lee W. D. (2015). Analysis of isokinetic muscle strength for sports physiotherapy research in Korean ssireum athletes. *J. Phys. Ther. Sci.* 27 3223–3226. 10.1589/jpts.27.3223 26644679 PMC4668170

[B61] O’Brien M. Mellalieu S. Hanton S. (2009). Goal-setting effects in elite and nonelite boxers. *J. Appl. Sport Psychol.* 21 293–306. 10.1080/10413200903030894

[B62] Olympics (2021a). *Archery.* Available online at: https://www.olympic.org/archery (accessed April 17, 2021).

[B63] Olympics (2021b). *Boxing.* Available online at: https://www.olympic.org/boxing (accessed April 17, 2021).

[B64] Olympics (2021c). *Judo.* Available online at: https://www.olympic.org/judo (accessed April 17, 2021).

[B65] Olympics (2021d). *Taekwondo.* Available online at: https://www.olympic.org/taekwondo (accessed April 17, 2021).

[B66] Olympics (2021e). *Wrestling.* Available online at: https://www.olympic.org/wrestling (accessed April 17, 2021).

[B67] Olympic Games. (2021). *Relive Past Games.* Available online at: https://olympics.com/en/olympic-games (accessed April 15, 2021).

[B68] Palmatier R. W. Houston M. B. Hulland J. (2017). Review articles: purpose, process, and structure. *J. Acad. Mark. Sci.* 46 1–5. 10.1007/s11747-017-0563-4

[B69] Papacosta E. Nassis G. P. Gleeson M. (2016). Salivary hormones and anxiety in winners and losers of an international judo competition. *J Sports Sci.* 34 1281–1287. 10.1080/02640414.2015.1111521 26584022

[B70] Park J. H. Shin S. H. (2015). The effect of university Kumdo Athletes self-management on life satisfaction and continued participation. *Korean J. Kumdo* 25 23–38.

[B71] Park J. K. (2000). Coping strategies used by Korean national athletes. *Sport Psychol.* 14 63–80. 10.1123/tsp.14.1.63

[B72] Park S. (2018). The effect of achievement goal orientation on the self-management of college judo athletes. *Asian J. Kinesiol.* 20 76–84. 10.15758/ajk.2018.20.4.76

[B73] Park S. M. Kwon T. D. Lee J. E. (2018). The relationship between the self-management strategy of Kendo players and exercise commitment and exercise satisfaction. *Injoma* 20 17–29. 10.35277/kama.2018.20.4.17

[B74] Phillips M. A. (2011). Classical martial arts training: a zen approach to health, wellness, and empowerment for women. *Can. Womens Stud.* 29 67–71.

[B75] Polaha J. Allen K. Studley B. (2004). Self-monitoring as an intervention to decrease swimmers’ stroke counts. *Behav. Modif.* 28 261–275. 10.1177/0145445503259280 14997952

[B76] Riemer H. A. Chelladurai P. (1998). Development of the athlete satisfaction questionnaire (ASQ). *J. Sport Exerc. Psychol.* 20 127–156. 10.1123/jsep.20.2.127

[B77] Sarkar M. Fletcher D. (2014). Psychological resilience in sport performers: a review of stressors and protective factors. *J. Sports Sci.* 32 1419–1434. 24716648 10.1080/02640414.2014.901551

[B78] Scanlan T. K. Carpenter P. J. Simons J. P. Schmidt G. W. Keeler B. (1993). An introduction to the sport commitment model. *J. Sport Exerc. Psychol.* 15 1–15. 10.1123/jsep.15.1.1

[B79] Schmidt R. A. Lee T. D. (2011). *Motor Control and Learning: A Behavioral Emphasis*, 5th Edn. Champaign, IL: Human Kinetics.

[B80] Schwab Reese L. M. Pittsinger R. Yang J. (2012). Effectiveness of psychological intervention following sports injury. *J. Sport Health Sci.* 1 71–79. 10.1016/j.jshs.2012.06.003

[B81] Seol J. H. Choi G. J. (2019). The effect of self-management of Taekwondo demonstration team members on exercise satisfaction and performance. *J. Martial Arts* 13 181–202. 10.51223/kosoma.2019.08.13.3.181

[B82] Son B. Cho Y. J. Jeong H. S. Lee S. Y. (2020). Injuries in Korean elite taekwondo athletes: a prospective study. *Int. J. Environ. Res. Public Health.* 17:5143. 10.3390/ijerph17145143 32708739 PMC7399793

[B83] Song E. I. Lee J. H. (2015). Effects of self-management of college Taekwondo athletes on sport confidence, exercise flow, and exercise satisfaction. *J. Korean Society Wellness* 10 171–182.

[B84] Strachan L. Munroe-Chandler K. (2006). Using imagery to predict self-confidence and anxiety in young elite athletes. *J. Imagery Res. Sport Phys. Activ.* 1 1–19. 10.2202/1932-0191.1004

[B85] Theodorakis Y. (1995). Effects of self-efficacy, satisfaction, and personal goals on swimming performance. *Sport Psychol.* 9 245–253. 10.1123/tsp.9.3.245

[B86] Theodorakis Y. Hatzigeorgiadis A. Chroni S. (2008). Self-talk: it works, but how? Development and preliminary validation of the functions of the self-talk questionnaire. *Meas. Phys. Educ. Exerc. Sci.* 12 10–30. 10.1080/10913670701715158

[B87] Tokyo 2020 (2020). *Archery.* Available online at: https://tokyo2020.org/en/sports/archery/ (accessed April 17, 2021).

[B88] Vesković A. Koropanovski N. Dopsaj M. Jovanoviæ S. (2019). Effects of a psychological skill training program on anxiety levels in top karate athletes. *Rev. Bras. Med. Esporte.* 25 418–422. 10.1590/1517-869220192505173969

[B89] Weinberg R. S. Gould D. (2015). *Foundations of Sport and Exercise Psychology*, 6th Edn. Champaign, IL: Human Kinetics Publishers.

[B90] Weiss W. M. Aloe A. M. (2019). Revisiting mediational models of sport commitment with female gymnasts. *Int. J. Sport Exerc. Psychol.* 17 600–616. 10.1080/1612197X.2018.1462228

[B91] Windsor G. (2018). *The Theory and Practice of Historical Martial Arts.* Spada Press.

